# Does an Overcrowded Emergency Department Reduce Moral Hazard? Lessons from Emergency Department Visits to Three Hospitals in an Israeli Metropolitan Area

**DOI:** 10.3390/healthcare10050915

**Published:** 2022-05-15

**Authors:** Fuad Basis, Aviad Tur-Sinai, Ziona Haklai

**Affiliations:** 1 Rambam Health Care Campus, Haifa 3109601, Israel; 2 Department of Health Systems Management, The Max Stern Yezreel Valley College, Yezreel Valley 1930600, Israel; avts2309@netvision.net.il; 3 Faculty of Medicine, Technion Israel Institute of Technology, Haifa 3525433, Israel; 4 School of Nursing, University of Rochester Medical Center, Rochester, NY 14642, USA; 5 Health Information Division, Ministry of Health, Jerusalem 9359102, Israel; ziona.haklai@moh.gov.il

**Keywords:** moral hazard, economy, non-urgent, emergency department, health policy

## Abstract

Metropolitan Haifa, Israel, has three hospitals: Rambam Health Care Campus, Bnai Zion Medical Center, and Carmel Medical Center. In 2007–2014, the length of stay at RHCC’s emergency department increased, while the number of visits decreased. We ask whether the increase in LOS is associated with the falling numbers of visits to other EDs, whether an increase in LOS induces more referrals to competing hospitals in the metropolitan area, and whether it pays to be a crowded ED in mitigating moral hazard. Average LOS at Rambam climbed from 3.5 h in 2000–2007 to 6.4 in 2008–2018. While the number of visits to Rambam decreased significantly, those to Bnai Zion increased significantly and quite linearly. A one-way ANOVA test reveals a statistically significant difference among the three hospitals. In addition, Rambam was significantly different from Carmel but not from Bnai Zion. When LOS stabilized at Rambam from 2016 to 2018 and increased at Bnai Zion, referrals to Rambam went up again. Policymakers should instruct all hospitals to publish LOS data, regulate referrals to EDs, and find an optimal LOS that will reduce competition, non-urgent visits, and moral hazard.

## 1. Introduction

Healthcare systems, medical insurance, and even emergency departments (EDs) are embroiled in an ongoing struggle against over-exploitation of EDs with non-urgent visits. In Israel, a report from the Ministry of Health (MOH) revealed that among 1.7 million visits to EDs countrywide, 68% occurred pursuant to referral by a physician, 22% came about due to accidents, about 17% were self-referrals, and the rest came from the hospitals’ own outpatient clinics. About 70% of patients admitted to EDs were discharged, that is, not admitted to hospitals’ regular departments [1].

The estimated percentage of non-urgent visits to EDs varies among countries and may be as low as 10.1%, as Honigman et al. found in a national study in the USA using a triage scale [2], and possibly as high as 64% in other countries such as Iran [3].

The variance may be attributed to differences in culture, healthcare systems, and community services. In any case, reducing non-urgent visits remains a challenge in every country, especially if physicians and citizens know that with a referral from a physician, ED visits are fully covered by medical insurance. Unfortunately, no study so far has examined the percent of non-urgent visits to any ED in Israel. 

Since 1995, all residents of Israel have had state health insurance by law and are entitled to a “basket” of free healthcare services. The country has four health-management organizations (HMOs) that started out historically as “health funds” and provide community service and hospitalization coverage to every resident by law. Each HMO is paid by the government on a capitation basis according to patient’s age and complexity. Concurrently, all HMOs must stay within an annual budget allocated to them by the state. To mitigate over-consumption of ED services, a patient who visits the ED without a referral by a physician, calls an ambulance, or fails to use his or her HMO’s night service clinic may be required to participate in payment for the visit and sometimes to foot the entire cost of treatment out of pocket (about USD 250). The purpose of these deductibles is to reduce non-urgent visits, the over-consumption of ED services, and the burden on EDs. It appears, however, that no specific study to measure the percentage of non-urgent visits in Israel has been undertaken.

During a thirty-day period in the 2006 Israel–Lebanon War, the number of visits to the Rambam Health Care Campus (RHCC) emergency department dropped by 30% because the hospital was under a barrage of missiles [4]. A similar percent was observed in the first two months of the COVID-19 outbreak as the population went into full lockdown for a full month in March 2020. By implication, perhaps, about 30% of ED visits may be non-urgent [5].

In metropolitan Haifa in Israel’s north, there are three public hospitals: RHCC, a level one trauma tertiary hospital; Bnai Zion Medical Center (B-ZMC), a municipal-government facility; and Carmel Medical Center (CMC), a hospital that belongs to the Clalit HMO, which provides about 55% of the population in Israel community services. The rest of the population gets its medical services and coverage from the other three HMOs. In 2007–2018, referrals to CMC’s ED were regulated and are largely controlled by the Clalit community clinics. ED visits to RHCC and B-ZMC are not limited in this manner. Namely, referrals to their EDs are not regulated by any HMO; the HMOs have no control over the number of visits to the ED and behave more like a “free market”.

RHCC, the central tertiary hospital in metropolitan Haifa, had 889 general beds with 59 in the ED in 2007 and 966 and 59, respectively, in 2018 (the start and end points of the study). B-ZMC had 401 general beds with 36 in the ED in 2007 and 431 and 43 ED, respectively, in 2018. CMC had 442 general beds with 42 in the ED in 2007 and 477 and 42, respectively, in 2018 [6]. Thus, although the number of the total hospital beds increased in all three hospitals, the number of ED beds in RHCC and CMC did not grow.

In RHCC in 2000–2007, the ED director, with the hospital manager’s backing, prevented the boarding of patients in the ED and limited the average length of stay (LOS) from admission or discharge or hospitalization to 3.5 h. In 2007, after both executives’ retirement, the new hospital manager chose a new director for the ED. In 2008, the ED temporarily went down to the basement for one year so that the original ED could be renovated and doubled in area. Within a few weeks, under the new manager, the length of stay (LOS) in the hospital wards started to increase gradually, the number of patients spending more than four hours in the ED began to rise, the ED became very crowded, the workload of ED medical staff increased, and the LOS in the ED also started to lengthen [7,8].

The newly renovated and enlarged ED building opened in 2009. According to some economic studies, a spacious facility may attract more consumers [9]. Indeed, the number of ED visits increased that year. In the next seven years (2009–2015), however, a paradoxical trend appeared: a gradual decline in visits to the ED, accompanied by a rise in LOS. From 2016 to December 2019 (before the first COVID-19 outbreak), the number of visits slanted upward, while LOS plateaued at 6.8 h on average (range: 6.2–7.6 h.).

At RHCC in 2018, there were 101,153 visits to the ED. Among these patients, 45.6% were referred by the triage nurse to the walk-in clinic within the ED. Most of these patients had triage scores of 4 and 5 according to the Canadian Triage Scale, which has been found to be a reliable scoring method in a meta-analysis study [10]. Only 3.2% of these walk-in clinic patients were admitted to the hospital departments as against 29.17% of admissions from the entire ED.

Trying to explain this paradox, we postulate that despite the renovated spacious ED at RHCC, there was a connection between the increase in LOS and the decrease in referrals to the ED. Patients and community physicians may have kept the continuously high LOS in mind when deciding to refer patients to any ED. Since the population of metropolitan Haifa did not decline during these years (2009–2018) and since any change in the HMOs’ behavior would have affected all hospitals in the metropolitan area, the authors assume that there may have been a spontaneous diversion of patients to the other two hospitals in the city.

Hence, the aim of this study is to see whether the combination of an overcrowded ED and high LOS may reduce non-urgent visits and, in turn, mitigate the moral hazard of ED over-utilization. The term “moral hazard”, in the context of healthcare systems, denotes additional healthcare coverage that people purchase when they take out health insurance. Health economists consider such coverage inefficient because it represents care that is worth less to consumers than it costs to produce [11]. Therefore, moral hazard is a longstanding concern among medical-insurance companies. Healthcare policymakers, too, have long been troubled by the stimulation of spending that health insurance causes although they are mindful that much of this expenditure is on costly healthcare that uninsured individuals could not otherwise afford [12].

The questions are: Is an increase in LOS associated with a decrease in the number of visits to EDs? Does an increase in LOS lead to an upturn in referrals to competing hospitals in the same metropolitan area? Furthermore, does it pay economically to be a crowded ED in reducing moral hazard?

## 2. Materials and Methods

### 2.1. Data Collection Procedure

This is a retrospective year series study that examined the number of visits to RHCC ED between 2007 and 2018. Concurrently, we examined the number of visits to the other general hospitals in the same city, B-ZMC and CMC, in the same period. The data for RHCC were harvested from the hospital’s business intelligence (BI) system; those for the other hospitals came from the Ministry of Health (MOH) using the same BI software.

RHCC is a tertiary referral hospital that provides level one trauma service for the north of Israel. The three hospitals combine serve about 570,000 residents in metropolitan Haifa (2018). The annual average increase in the metropolitan Haifa in 2007–2018 was 1.3% [13].

### 2.2. Measures and Data Analysis 

All data were analyzed using the SPSS software v 27.0. Data on the average number of visits to each ED were collected on a quarterly basis from 2007 to 2018 in order to make it statistically applicable (Table 1). Data on the average LOS in three hospitals were harvested collected from the MOH software. Since the comparison of averages comprises a large number of visits, the year-to-year delta (Δ) change in the number of visits was calculated and compared in absolute numbers (positive integers) using the one-way ANOVA test. To measure a positive or a negative correlation among the three hospitals in the year-to-year change in the number of visits, if such occurred, we used the Pearson correlation coefficient to see if the decline in the number of visits to RHCC’s ED was correlated with any change in visits to the other two hospitals.

## 3. Results

**Figure 1:**
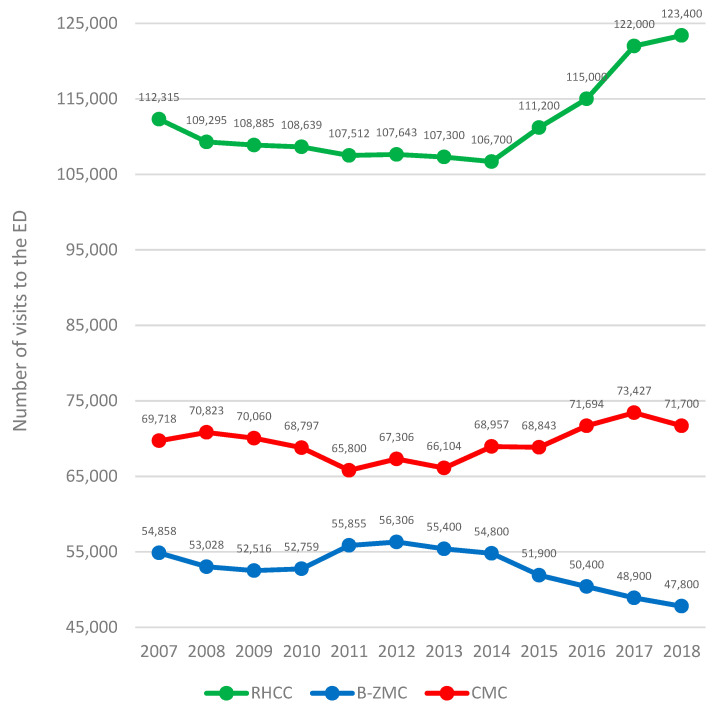
Trendline of visits to EDs at three hospitals in metropolitan Haifa, 2007–2018 (Rambam Health Care Campus (RHCC), Bnai-Zion Medical Center (B-ZMC), and Carmel Medical Center (CMC)).

**Figure 2:**
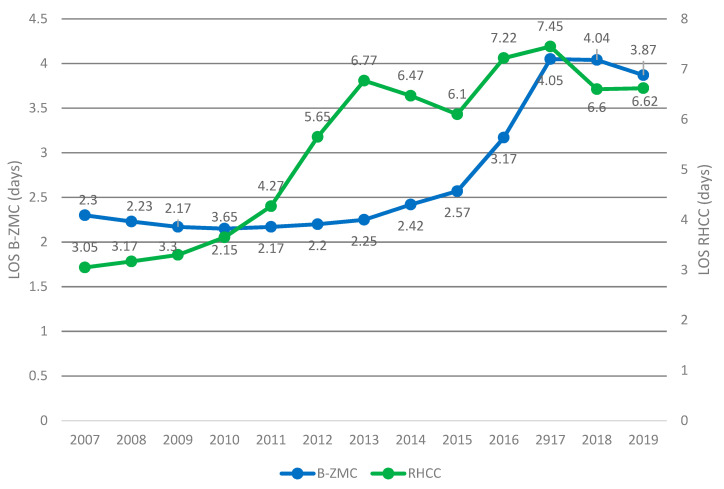
Patients’ length of stay (workup time) until decision is made (presented on a double vertical axis: left, B-ZMC; right, RHCC).

Together with the decrease in the number of visits to the RHCC ED (from 2007 until 2014), there was an increase in visits to the B-ZMC ED, and when the trend reversed direction in 2015, the upturn in visits to RHCC’s ED was countered by a decrease in visits to B-ZMC’s ED. At CMC, however, visits to the ED rose gently and gradually, possibility reflecting population increase in metropolitan Haifa (Figure 1). Parallel to the prolongation of LOS at RHCC’s ED, there was an increase in the burden in B-ZMC’s ED and a gradual rise in LOS, perhaps for the reason mentioned previously (Figure 2). The CMC ED may not have been affected because the number of visits is controlled and regulated by Clalit’s community clinics; therefore, we omitted it from Figure 2 in order to focus on the two governmental medical centers (see discussion below).

Applying the Pearson correlation test, we found a very significant and quite linear negative correlation in the number of ED visits at RHCC as against B-ZMC (r (12) = −0.9119, *p* < 0.001). Concurrently, referrals to the ED of CMC, the Clalit hospital, to which the number of referrals by the HMO’s physicians is regulated, showed a positive weak correlation between RHCC and CMC (r (12) = 0.4181, *p* = 0.003) (Figure 1). This may indicate the existence of a rather strong linear inverse correlation between RHCC and B-ZMC in regard to the number of visits to the ED; when the number of visits to RHCC’s ED raised or decreased, the upturn had no direct effect on the number of referrals to CMC’s ED. In other words, when the number of visits to the RHCC ED decreased, visits to B-ZMC’s ED increased and vice versa. The Pearson correlation test between B-ZMC and CMC, however, was negative and statistically significant, (r (12) = −0.8483, *p* < 0.001). This may indicate that the overall trend of visits to the ED at B-ZMC was remarkable and correlated inversely to visits to the EDs of both CMC and RHCC. To some extent, the figure shows an increase in total referrals to all three EDs in Haifa. While the number of visits to RHCC was declining, however, visits to CMC increased but were more inversely correlated to B-ZMC.

Comparing differences (increase or decrease) in the absolute numbers of visits to the ED between each year and the year before (year to year) on a quarterly basis from 2007 to 2018 using the one-way ANOVA test, we found a statistically significant difference among the three hospitals (F (3, 31) = 3.822, *p* = 0.032, η2 = 0.18). Post hoc comparisons using the Tukey HSD test indicated that the mean scores in the year-to-year difference, in absolute numbers, for RHCC (M = 2915, SD = 2136) were significantly different from those of CMC (M = 1353, SD = 960) but not different from B-ZMC (M = 1638, SD = 923). This may indicate that the year-to-year change in absolute number of visits is correlated between RHCC and B-ZMC but not with that of CMC, to which referrals are regulated by Clalit’s clinics.

## 4. Discussion

Parallel to the increase in LOS at RHCC ED despite the construction of a spacious new ED, there was a gradual and correlated decline in the number of visits. On the other hand, visits to the ED of the B-ZMC municipal-government hospital increased. As the burden on B-ZMC’s ED grew, so did LOS. At CMC, where visits are regulated by the Clalit HMO, there was a moderate gradual increase in visits, perhaps reflecting population growth in metropolitan Haifa.

In another phenomenon observed, from January 2016 to December 2019 (preceding the first COVID-19 outbreak) as LOS at RHCC’s ED reached a plateau (6.2–7.6 h), a gradual reversion to increase in the number of visits was observed; it was correlated with a decline in visits to B-ZMC’s ED. The number of year-to-year visits to CMC ED, however, did not change significantly. This phenomenon somewhat clashes with the trend from 2007 to 2015. The explanation may be found in the science of economics: It is clear that when the price of an essential product increases significantly, its consumption decreases, and the consumption of a cheaper (inferior) competing product (such as butter and margarine) rises; later on, so does its price. Thus, LOS at the B-ZMC ED may have been prolonged due to strong demand. Perhaps time really is money for some patients. However, if the product is essential, as in the case of lemons in Winand’s paper [14], and its cost rises, the consumption and the price of the counter product (lemon salt) rises. Since lemons remain essential, and their price stabilizes (as happened in LOS at RHCC from 2016 to December 2019) within a certain time, we expect people get used to the constant higher price and consumption of the good or service will gradually rebound to its previous level [14,15].

RHCC is a level one trauma center that meets of needs of patients in all specialties. Therefore, it may be considered an “essential” product in terms of economics. When its LOS time doubled, citizens may have preferred the “competing product” (the margarine). However, when LOS stabilized at RHCC and lengthened at B-ZMC (as the cost of lemons rose), citizens made a gradual comeback, as they got used to the stable high LOS (similar to the price in the case of the original lemons).

Non-urgent visits remain a concern among medical-insurance companies. Nipkay et al. showed that, in the context of ED visits, the expansion of Medicaid also increased the consumption of ED services in the USA, consistent with the results of polls among emergency physicians [16]. The definition of a non-urgent visit remains somewhat problematic and differs from one country to another, perhaps depending, among other things, on the quality of community health services, the medical insurance system, and the population [17,18]. In Israel, as mentioned above, the healthcare costs of all residents are equally covered by national health insurance. Patients who refer themselves to a hospital at a time of day when community clinics are open may have to pay all ED expenses out of pocket upon discharge. Although the percentage of patients in metropolitan Haifa who visit EDs without a referral from a community physician has been quite constant in the past three years (about 17% in average) [6], the average percentage of patients discharged from RHCC’s ED in the past five years is 70.4%.

Since some 55% of citizens in metropolitan Haifa receive service at the Clalit HMO community clinics, these clinics from time to time are instructed to regulate patients’ referral to CMC, which is owned by the same HMO. The other EDs in Haifa do not have this privilege; the other HMOs’ community clinics may refer patients to the three hospitals according to their needs, hospital proximity, or perhaps waiting time (LOS) in the ED. In an attempt to regulate referrals to its ED, RHCC publishes the average LOS of patients at the ED on an hourly basis on its website [19], allowing citizens to know the “burden” at this ED at any given moment. No other hospital in the Haifa District publishes this information. Our study suggests that LOS at the ED may play a role in patients’ referral to hospitals.

From an economic point of view, it seems that it pays to be a busy ED. From the medical standpoint, too, this may be true to a certain limit, as studies have shown that ED overcrowding and patients spending hours in the ED waiting for an empty bed in the hospital wards may lead to increase in morbidity and mortality [20,21]. On the other hand, it has been found that patients boarded in the ED consume more time than new patients [22]. Therefore, there may be a conflict of interest between the Ministry of Health and the Ministry of Finance. The Ministry of Health wishes to maximize efficiency and rewards hospitals for shortening LOS in the ED; the Ministry of Finance is interested in reducing healthcare-system expenditure by mitigating overuse of hospitals’ EDs services.

Although patients in Israel do not pay for medical services directly, they seem willing to substitute money for time. Our study may show that LOS may affect patients’ choice of ED. If such is the case, controlling LOS may affect ED consumption. There may be a point where hospitals’ cost and benefit match, as is the case in the consumption curve in economics. At such a point, LOS is long enough to reduce non-urgent visits but short enough to prevent harm to the quality of care. From patients’ viewpoint, there may be a point on the consumption curve where, beyond a certain LOS, they may think twice before referring themselves to the ED. A game between consumer and supplier appears to be taking place. At this point, a Nash equilibrium [23] in game theory may exist in the healthcare system “market” even though healthcare systems behave as an unequal market [24]. Some suggest breaking the Nash equilibrium by reorganizing acute stroke care via cooperation and coordination of activities among hospitals in order to attain better care [25]. By cooperating, “producers” may be more successful in controlling consumers’ measures, thus allowing the possibility of better healthcare. Although such a practice may aggravate inequality in the healthcare market, it would create a win-win situation by causing patients to be referred to hospitals that will treat them in time. Hence, there seems to be reason to consider interfering with the natural equilibrium and biasing it in favor of hospitals’ needs (reducing non-urgent visits and mitigating the burden) without harming patients’ well-being. Although coordinating LOS among EDs may create something of a monopoly, it may give EDs more control over their situation. Therefore, if there is a Nash equilibrium in healthcare services, it would be beneficial to control it by adding a positive or negative incentive to deviate from the initial strategy.

In this study, we elicited a hypothetical theory from our observation of what occurred in three hospitals that served the same population for more than ten years. Our study raises more questions than it answers on the health-economics side, setting the background for further research that would produce a mathematical model for the regulation of the burden on EDs and the decrease in non-urgent visits from the perspective not of the emergency medical system of healthcare-system policy in general.

In Israel, the Ministry of Health rewards the three EDs countrywide that have the shortest LOS. According to our findings, competition for this incentive may encourage patients to refer themselves to specific EDs LOS at B-ZMC, for example, which is half of that at RHCC. MOH should ask all hospitals to publish their LOS data on their websites as RHCC does. In case of ED overburden, MOH policymakers can intervene by diverting ambulances and instructing community physicians in the same metropolitan area via the heads of their HMOs, thus equalizing LOS in all EDs. The authors assume that at a certain equilibrium (an optimal LOS), patients who have non-urgent cases may postpone or cancel their visits to the ED. Fast-track evaluation may exacerbate the moral hazard, while a very high LOS may impair the quality of care. Once policymakers consider this proposal, a new study to confirm our hypothesis would be worth performing.

As noted above, the share of patient visits to EDs in Israel without referral by community physicians has approximated 17% in the past three years. However, about 70% of patients who visit RHCC’s ED and more that 97% of patients in the walk-in clinic are discharged. Therefore, some may claim that community physicians refer patients to EDs more than needed. We suggest the application of better control over community physicians’ judgment about when to refer patients to EDs. It may be worth considering charging patients a small or symbolic fee even if they are referred to ED by community physicians. Once patients are admitted to a regular hospital department or a specific intervention at the ED is found necessary, the fee would be refunded. This may cause patients to think twice about needing a referral to the ED and cause physicians to bear in mind that their patients may have to cover some of the cost of their ED referrals of the ED visit is considered non-urgent.

## 5. Limitations of the Study and Suggestions for Future Research

The main strengths of this study are its administrative data and its reliance on inputs such as unique datasets that yield a broader and more significant view of the phenomenon in question. However, some limitations should be noted. This is an observational time series study conducted only in one major metropolitan city in Israel. The findings presented, along with the contents of the literature on influencing the healthcare market and mathematical and economic theories, may inspire some to calculate a better balance between the optimal LOS in EDs and ED services consumption. This may be of interest to government policymakers. Some studies set the optimal LOS at four hours and define the maximum at six hours [26]. These times are longer than at B-ZMC and shorter than those in RHCC. Increasing LOS at all hospitals in the same city may reduce non-urgent visits without harming healthcare quality. Furthermore, the authors believe that publishing the LOS on hourly basis, as is done at RHCC, may have a “Waze navigator effect” on diverting and directing patients to less-crowded EDs. We suggest the policymakers should consider this option in order to regulate patient referrals, as the Clalit HMO does for its hospitals. In addition, since the three public hospitals in this study serve the same community, and any demographic change in this community would apply to all of them, it stands to reason that the changes were not biased by an external factor because the status quo was maintained during all these years. However, there may be others factor that are unknown or have not been considered.

Another limitation of the study is the assumption that the inverse correlation between the number of referrals to RHCC ED and B-ZMC ED is causally related. However, other variables unknown to the authors may play a role although we did not anticipate environmental changes that, to the best of our knowledge, may include additional variables.

Since this study was done before the COVID-19 pandemic, we propose a future study in which the trend that we examined may be investigated in the post-pandemic period. During the pandemic, COVID-19 patients were routed and coordinated among hospitals with the help of the Ministry of Health in order to regulate the load of hospitalizations. We propose to examine whether this policy of the Ministry of Health and the hospitals affected the regulation of referrals of regular patients to hospitals in the same district after the regulation policy was adopted. Such a study might be able to show whether there are lessons to be learned from the treatment of patients with COVID-19 for other non-COVID-19 patients once the pandemic subsides. In conclusion, our study describes the referrals of patients to three hospitals’ EDs in the same district in an attempt to analyze the behavior of patients in terms of the “healthcare market”. By understanding people’s behavior regarding the effect of waiting time from admission to discharge from the ED, and building software that can estimate the load in each ED and LOS in real time, the Ministry of Health may be able to divert patients from one ED to another in order to regulate LOS for an optimal time value that can reduce non-urgent visits without compromising the quality of care [27,28]. In addition, by releasing these data to community physicians, citizens, and ambulance services in real time, these players may acquire the option of diverting patients from one hospital to another according to the existing load in each hospital’s ED or, perhaps, to postpone non-urgent visits to another date or even cancel them.

## Figures and Tables

**Table 1:** Descriptive data on number of visits to three hospitals’ emergency departments (R, RHCC; B, B-ZMC; C, CMC). N = number of year quarters measured.

Hospital	N	Mean	Std. Deviation	Std. Error	95% Confidence Interval for Mean		Minimum	Maximum
					Lower Bound	Upper Bound		
RHCC	13	114,129.92	7349.784	2038.463	109,688.49	118,571.35	108,300	130,800
B-ZMC	13	52,378.62	3190.956	885.012	50,450.34	54,306.89	46,400	56,306
CMC	13	69,602.23	2286.149	634.064	68,220.72	70,983.74	65,800	73,427
Total	39	78,703.59	26,771.140	4286.813	70,025.39	87,381.79	46,400	130,800

## Data Availability

The data presented in this study are available on request from the corresponding author. The data are not publicly available due to that the research data re not openly published.
